# Dietary oleic acid contributes to the regulation of food intake through the synthesis of intestinal oleoylethanolamide

**DOI:** 10.3389/fendo.2022.1056116

**Published:** 2023-01-17

**Authors:** Miki Igarashi, Kensuke Iwasa, Tetsuhiko Hayakawa, Tsuyoshi Tsuduki, Ikuo Kimura, Kei Maruyama, Keisuke Yoshikawa

**Affiliations:** ^1^ Advanced Clinical Research Center, Institute of Neurological Disorders, Kawasaki, Japan; ^2^ Department of Pharmacology, Faculty of Medicine, Saitama Medical University, Saitama, Japan; ^3^ Department of Applied Biological Science, Graduate School of Agriculture, Tokyo University of Agriculture and Technology, Tokyo, Japan; ^4^ Department of Bioscience and Biotechnology for Future Bioindustries, Graduate School of Agricultural Science, Tohoku University, Sendai, Japan; ^5^ Laboratory of Molecular Neurobiology, Graduate School of Biostudies, Kyoto University, Kyoto, Japan

**Keywords:** oleic acid, oleoylethanolamide (OEA), satiety, intestine, food intake

## Abstract

**Introduction:**

Among the fatty acid ethanolamides (FAEs), oleoylethanolamide (OEA), linoleoylethanolamide (LEA), and palmitoylethanolamide (PEA) are reported to be involved in feeding regulation. In particular, OEA is well characterized as a satiety signal. Following food consumption, OEA is synthesized from oleic acid (OA) *via* an *N*-acyl phosphatidylethanolamine-specific phospholipase D-dependent pathway in the gastroenterocytes, and OEA induces satiety by recruiting sensory fibers. Thus, we hypothesized that dietary OA is an important satiety-inducing molecule. However, there has been no direct demonstration of the effect of dietary OA on satiety induction without the influence of the endogenous biosynthesis of OA from stearic acid (SA) or other FAEs.

**Methods:**

In this study, we used two experimental diets to test our hypothesis: (i) an OA diet (OAD; 38.4 mg of OA/g and 7.2 mg of SA/g) and (ii) a low OA diet (LOAD; 3.1 mg of OA/g and 42.4 mg of SA/g).

**Results:**

Relative to mice fed the OAD, mice fed the LOAD for two weeks exhibited reduced levels of jejunal OEA but not jejunal LEA and PEA. The LOAD-fed mice showed an increase in food intake and body weight gain. Moreover, LOAD-induced increase in food intake was immediately observed after the switch from the OAD, whereas these effects were diminished by the switch back to the OAD. Furthermore, treatment with OA and OEA diminished the effects of LOAD on food intake.

**Conclusion:**

Collectively, these results show that dietary OA is a key factor in the reduction of food intake and increase in satiety mediated by OEA signaling.

## 1 Introduction

Oleoylethanolamide (OEA), one of fatty acid ethanolamides (FAEs), is known to control satiety and lipid metabolism (1–4). OEA is generated in the enterocytes during the digestion of food and functions through its activation of peroxisome proliferator-activated receptor-α (4, 5). To induce satiety, OEA recruits the sensory afferents of the vagal nerve (1).

OEA can be derived directly from diet or synthesized from oleic acid (OA) in the gastrointestinal enterocytes (1, 5–7). Free OA is internalized into the intestinal enterocytes through the action of the membrane-localized fatty acid translocase CD36 and incorporated into the *sn*-1 position of phosphatidylcholine during the digestion of food (2). The OA incorporated into phosphatidylcholine is then transferred to the amine group of phosphatidylethanolamine *via* the action of *N*-acyl transferase (8), resulting in the generation of *N*-oleoyl-phosphatidylethanolamine (NOPE). OEA is released from NOPE through enzymatic hydrolysis with an *N*-acyl phosphatidylethanolamine-specific phospholipase D (NAPE-PLD) (5, 9, 10). In addition, OEA is synthesized *via* NAPE-PLD-independent multistep pathways that involve several enzymes, including glycerophosphodiester phosphodiesterase 1 and α/β-hydrolase domain–containing protein 4 (5, 9, 10). Other FAEs are also synthesized through the same pathways. Linoleoylethanolamide (LEA) and palmitoylethanolamide (PEA) are known to be involved in the reduction of food intake, while the endocannabinoid anandamide (AEA) is known to induce appetite (11, 12).

Refeeding after food deprivation is known to stimulate the increase of OEA in the guts of lean rodents, Burmese python (*Python molurus*), and goldfish (*Carassius auratus*) (5, 6, 13, 14). In addition, the intraduodenal infusion of dietary lipids containing OA increases jejunal OEA levels in lean rodents, whereas infusions of protein or carbohydrate do not increase these levels (6, 7). Therefore, dietary OA is an important factor to regulate OEA signaling in the gut. It is also possible that OA synthesized from stearic acid (SA) is converted into OEA because SA is contained in dietary fat and intestinal stearoyl-CoA desaturase is expressed in the intestine (15).

In the present study, we investigated whether dietary OA levels contribute to the biosynthesis of intestinal OEA and food intake in mice, excluding the influence of other FAEs such LEA and PEA, and endogenously synthesized OA. To this end, we fed mice two experimental diets: (i) an OA diet (OAD; 38.4 mg of OA/g diet and 7.2 mg of SA/g diet) and (ii) a low OA diet (LOAD; 3.1 mg of OA/g diet and 42.4 mg of SA/g diet) (16, 17). Each diet contained similar levels of essential fatty acids; linoleic acid (12 mg/g diet) and α-linolenic acid (2 mg/g diet) (18, 19). We found that dietary OA levels control food intake through the synthesis of OEA without the influence of other anorexic FAEs and endogenously synthesized OA.

## 2 Materials and methods

### 2.1 Animals

C57BL/6J mice were obtained from Japan SLC Inc. (Hamamatsu, Japan) and acclimated to the animal facility for one week. During acclimation, mice were fed CLEA Rodent Diet CE-2 (CLEA Japan, Inc., Tokyo, Japan) and housed under controlled conditions: 20°C–25°C, 40%–60% humidity, and a 12/12 h light/dark cycle. Animal experiments were conducted in accordance with the guidelines of the Committee on the Ethics of Animal Experiments of Saitama Medical University and the Tokyo University of Agriculture and Technology, and the experiments were approved by the Animal Research Ethics Subcommittees (Saitama Medical University permit number 3190; Tokyo University of Agriculture and Technology permit number 28–87).

### 2.2 Diet

The dietary compositions of the OAD and LOAD are shown in **Table 1**. These diets were created based on the AIN-93G formulation with modifications to the fatty acid content (20). The diets contained 7% fat, and several dietary oils were mixed to modify the fatty acid composition of each diet. OAD contained hydrogenated soybean oil, olive oil, high–linoleic acid safflower oil, and linseed oil, whereas LOAD contained hydrogenated soybean oil, high–linoleic acid safflower oil, and linseed oil. These diets were prepared by Oriental Yeast Co., Ltd. (Tokyo, Japan).

**Table 1:** Ingredient composition of experimental diets.

	OAD	LOAD
Ingredients	g/100g	g/100g
Casein	20.0	20.0
L-Cystine	0.3	0.3
Starch	49.7	49.7
Dextrinized cornstarch	13.2	13.2
Sucrose	0.0	0.0
Hydrogenated soybean oil	0.7	5.2
Olive oil	4.9	0.0
High linoleic acid-Safflower oil	1.1	1.5
Linseed oil	0.4	0.4
Cellulose powder	5.0	5.0
AIN93G Mineral mix	3.5	3.5
AIN93 Vitamin mix	1.0	1.0
Choline bitatrate	0.3	0.3
TBHQ	0.001	0.001
Total	100	100

The fatty acid content (mg/g) of the diets is shown in **Table 2**. The fatty acids in the diets were measured using gas chromatography as described below. The OAD contained OA at 38.4 mg/g diet, whereas the LOAD contained OA at 3.1 mg/g diet (16, 17). Both diets contained similar and adequate levels of linoleic acid and α-linolenic acid (18, 19).

**Table 2:** Fatty acid composition of experimental diets.

	OAD	LOAD
	mg/g	
12;0	0.037 ± 0.003	0.031 ± 0.001
14;0	0.148 ± 0.011	0.146 ± 0.006
14:1(n-5)	0.050 ± 0.004	0.034 ± 0.001
16:0	7.1 ± 0.5	3.6 ± 0.1
16:1(n-7)	0.46 ± 0.03	0.049 ± 0.002
18:0	7.2 ± 0.3	42.4 ± 1.4
18:1(n-9)	38.4 ± 3.0	3.09 ± 0.11
18:1(n-7)	1.27 ± 0.10	0.16 ± 0.01
18:2(n-6)	12.7 ± 1.0	11.0 ± 0.5
18:3(n-6)	ND	ND
18:3(n-3)	2.2 ± 0.2	1.7 ± 0.1
20:0	0.35 ± 0.02	0.86 ± 0.03
20:1	0.20 ± 0.02	0.06 ± 0.00
20:2	ND	ND
20:4	ND	ND
22:0	0.11 ± 0.00	0.23 ± 0.01
22:6	ND	ND
Total	70.1 ± 5.2	63.4 ± 2.2

Values are expressed as the mean ± SEM. n = 4. ND, not detected.

### 2.3 Experimental design

#### 2.3.1 Effects of two- or fifteen-week LOAD feeding periods

Four- or seven-week-old male mice were acclimatized to our animal facility for one week. Eight-week-old mice were fed the OAD or LOAD for two weeks, and the mice were housed in a measurement system to assess their energy metabolism and locomotor activity at day 11 of feeding. Their food intake and body weight were measured every 2–3 days. At day 14, the mice were fasted for 5 h. Thereafter, the blood glucose of the mice was measured using a portable glucometer with compatible glucose test strips (OneTouch R Ultra R, LifeScan Inc., Milpitas, CA, USA), after which they were anesthetized using isoflurane (Pfizer Inc., New York, USA). Blood was collected from the inferior vena cava using heparinized tubes, and plasma was separated *via* centrifugation (7,000 *g* for 5 min at 4°C). Mouse tissues were harvested and weighed, and collected samples were stored at −80°C. Five-week-old mice were fed the OAD or LOAD for 15 weeks, and their body weight was measured weekly. At week 15, the food intake in 24 h was measured for individual mice, after which the mice were sacrificed for tissue harvesting.

#### 2.3.2 Cyclic OAD and LOAD feeding

Six-week-old male mice were purchased and acclimatized to our animal facility for one week. They were fed the OAD for one week and then divided into two groups. Group A was fed the OAD for two weeks, whereas group B was fed the LOAD for one week and then switched to feed on the OAD for one additional week. Food intake during dark periods was measured on days 1, 3, 7, 8, 10, and 14.

#### 2.3.3 Effects of OA and OEA treatments on LOAD feeding

Seven-week-old mice fed the OAD for one week were prepared, after which they were treated with fatty acids and OEA 30 min before the dark cycle began. The mice were then fed the OAD or LOAD during the dark periods, and their food intake was measured. Fatty acids, i.e., OA and palmitic acid (PA) (both obtained from Nu-Chek Prep Inc., Elysian, MN, USA), were dissolved in phosphate-buffered saline (PBS) containing 0.3% xanthan gum (100 mg of fatty acid/0.5 mL per mouse) for oral gavage treatment. OEA was dissolved in 10% dimethyl sulfoxide (DMSO; Sigma-Aldrich Co. LLC, St. Louis, MO, USA) with 3% xanthan gum (Tokyo Chemical Industry Co., Tokyo, Japan) in PBS for the oral treatment (5 mg of OEA/200 μL per mouse), whereas OEA was dissolved in 70% DMSO in PBS (0.5 mg of OEA/50 μL per mouse) for the intraperitoneal treatment.

#### 2.3.4 Effect of OAD and LOAD refeeding after food deprivation

Six-week-old male mice were acclimatized to the animal facility for one week and then fed the OAD for one week. Subsequently, the mice were deprived of food for 24 h, after which they were refed either the OAD or LOAD for 1 h. During the refeeding period, food intake was measured. Finally, the jejunal of mice were collected for OEA analysis.

### 2.4 FAE and FA analyses

Tissues or food were homogenized in methanol (1 mL) containing certain amounts of the internal standard [d^4^-OEA (Cayman Chemical Co., Ann Arbor, MI, USA) for FAE analysis; triheptadecanoin (Nu-Chek) for fatty acid analysis in food and tissue]. The homogenates were then added to chloroform (2 mL) and 0.5 M of KCl (0.75 mL) to extract the total lipids. The organic phases were collected and dried using a nitrogen gas stream. Total lipids were dissolved in 60 µL of a solvent mixture of chloroform and methanol (1:3, v/v) for FAE analysis, or in 0.5 mL of methanol for fatty acid analysis. The total lipid extracts in 0.5 mL of methanol were mixed with 0.5 mL of 0.2% H_2_SO_4_-MeOH and heated at 70°C for 3 h to undergo methylation.

FAE analyses were performed following a method described previously (21) using a Nexera UHPLC system consisting of a LCMS-8040 triple quad mass spectrometer equipped with an electrospray ionization interface (Shimadzu Corp., Kyoto, Japan). Briefly, FAEs were separated on a ZORBAX Eclipse XDB-C18 column (2.1 × 100 mm, 1.8 μm; Agilent Technologies, Santa Clara, CA, USA) under an acetonitrile [FUJIFILM Wako Pure Chemicals Corp. (Wako), Osaka, Japan] gradient condition. Individually optimized multiple reaction monitoring parameters were used for both the target compounds and internal standards. The absolute levels of FAEs were quantified using a calibration curve [OEA, PEA, AEA, LEA, and stearoylethanolamide (SEA); Cayman].

Fatty acid analysis was performed following a method described previously (21). Prepared fatty acid methyl esters were analyzed using gas chromatography (GC-4000 Plus, GL Sciences, Tokyo, Japan) with a flame ionization detector and a SUPELCOWAX 10 fused silica capillary column (60 m × 0.25 mm, 0.25 µm film thickness; Supelco, Bellefonte, PA, USA) (21).

### 2.5 Analysis of energy metabolism and locomotor activity

The respiratory quotient, energy expenditure, and locomotor activity of mice fed either OAD or LOAD were measured. At day 10 during two weeks of feeding, mice were individually housed in a measurement chamber (LP-80CCFL-8AR; Nippon Medical & Chemical Instrument, Co. Ltd., Osaka, Japan) and acclimatized for at least 12 h. The oxygen consumption (VO_2_) and carbon dioxide production (VCO_2_) of the mice were measured using an MK-5100MS device (Muromachi Kikai Co. Ltd.), and their locomotor activity was measured using SUPERMEX PAT.P (Muromachi Kikai Co. Ltd.). The conditions in the measurement chamber were controlled as follows: 12/12 h light/dark cycle, 22°C, and 30%–60% humidity. Measurements were taken for 48 h, including the acclimation period (≥12 h). The mice had free access to food and water. Respiratory quotients and energy expenditure were calculated based on the VO_2_ and VCO_2_ values recorded, whereas locomotive activity was the number of cross counts of thermal radiation by mouse.

### 2.6 Other analyses

Plasma triacylglycerol and total cholesterol levels were determined using a LabAssay™ Triglyceride Kit (Wako) and a LabAssay™ Cholesterol (Wako), respectively. To analyze fecal fatty acids, the total lipids in feces were extracted as described above without adding an internal standard, after which they were saponified using 0.3 N of NaOH (Wako) in 90% MeOH (Wako). The fatty acids were extracted using n-hexane (Wako), and the organic layer was dried using N_2_ gas. The residues were dissolved in 2-propanol (Wako), and fatty acid concentrations were measured using a LabAssay™ NEFA (Wako).

### 2.7 Statistical analysis

All measured values are expressed as means ± standard errors of the means (SEMs). GraphPad Prism version 7.04 (GraphPad Software Inc., La Jolla, CA, USA) was used to conduct the statistical analysis. Statistical significance was evaluated using unpaired Student’s t-tests, one-way ANOVA, or two-way ANOVA depending on the data. *Post-hoc* analyses were conducted using Tukey’s multiple comparison test to compare means when significant differences were identified. Differences between groups were considered significant when P < 0.05.

## 3 Results

### 3.1 Effects of LOAD feeding for two weeks

**Figure 1:**
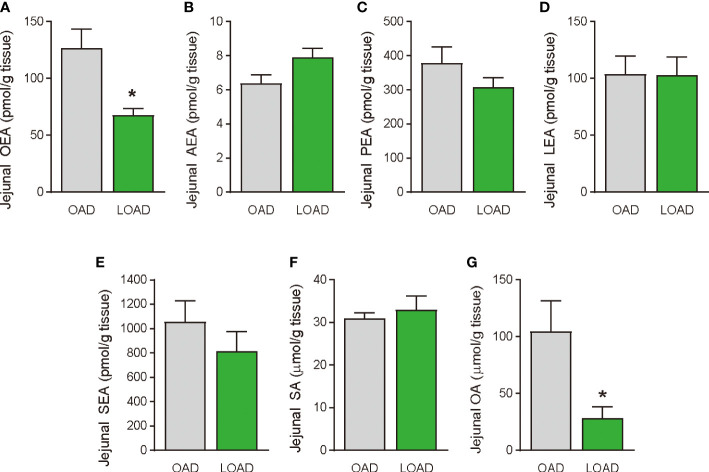
Jejunal FAE levels in mice fed the OAD and LOAD for two weeks. Mice were fed the OAD and LOAD for two weeks and jejunal FAEs were measured using LC–MS/MS. Jejunal SE and OA were measured using GC/ESI. **(A)** OEA, **(B)** AEA, **(C)** PEA, **(D)** LEA, **(E)** SEA, **(F)** SA, and **(G)** OA. Values are expressed as means ± SEMs (n = 5). *P < 0.05.

**Figure 2:**
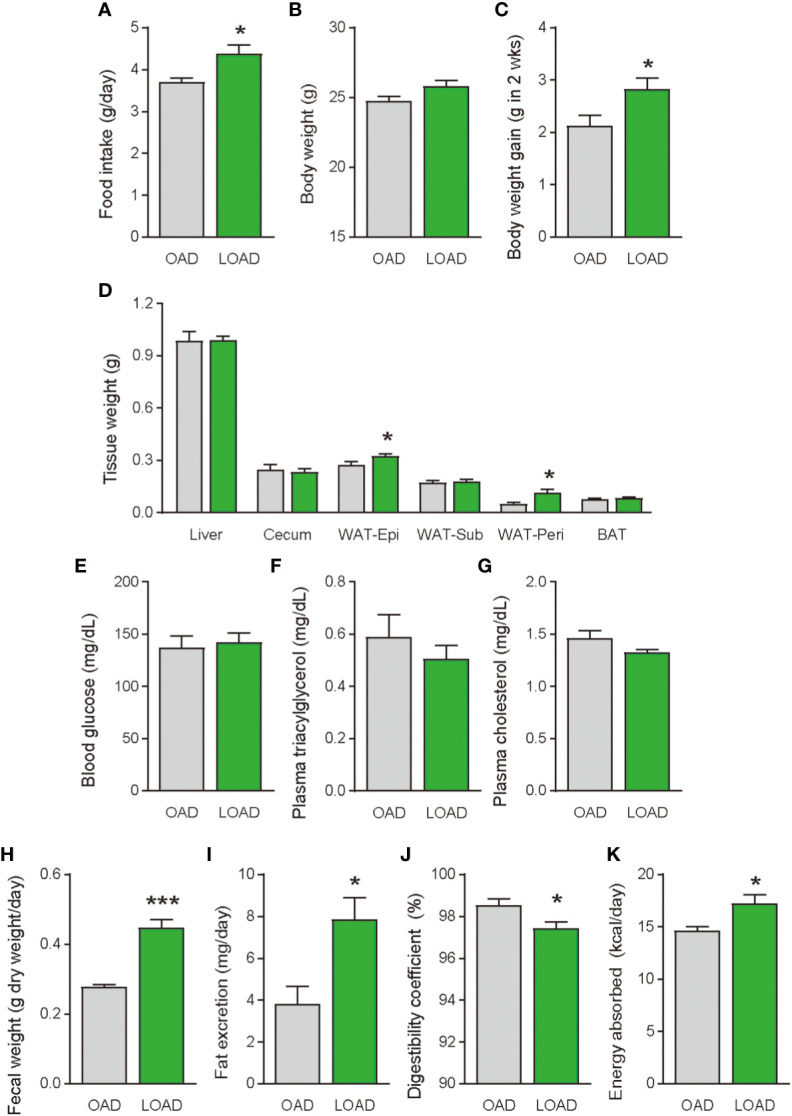
Food intake, body weight gain, tissue weight, and blood glucose, plasma lipid levels, and fecal fat excretion in mice fed the OAD and LOAD for two weeks. **(A)** Food intake was measured on the last day of the feeding period (n = 5 for each group). **(B)** Final body weight on the last day of the feeding period and **(C)** body weight gain during the feeding period in mice fed the OAD and LOAD (n = 8 for each group). **(D)** Weights of the liver, cecum, epidydimal adipose tissue (WAT-Epi), subcutaneous adipose tissue (WAT-Sub), peritoneal adipose tissue (WAT-Peri), and brown adipose tissue (BAT), **(E)** blood glucose levels, **(F)** plasma triacylglycerol levels, and **(G)** plasma total cholesterol levels of mice the OAD and LOAD (n = 5–9). Feces were collected for 24 h on the final day of feeding. **(H)** Fecal weight of feces collected over 24 h. **(I)** Total lipids were extract from the feces and hydrolyzed. Extracted fatty acids were measured, and the values are expressed as triacylglycerol levels. **(J)** Digestibility coefficients were calculated using fat excretion (mg/mouse) and fat consumption (mg/mouse) in individual mice. **(K)** Energy absorption was calculated using fat excretion and total energy consumption. Values are expressed as means ± SEMs (n = 5-9). *P < 0.05; ***P < 0.001.

**Figure 3:**
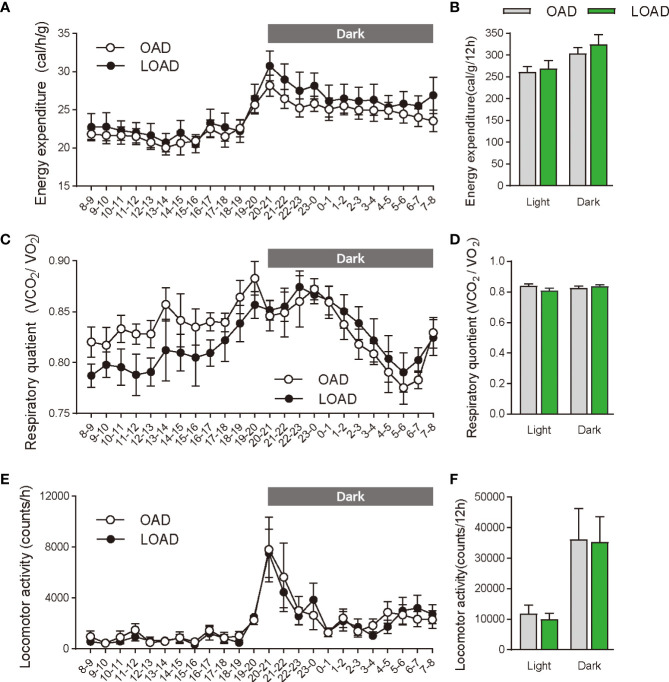
Energy expenditure, respiratory quotient, and locomotor activity in mice fed the OAD and LOAD for two weeks. **(A, B)** Energy expenditure, **(C, D)** respiratory quotient, and **(E, F)** locomotive activity were measured in mice on day 12 of feeding on either the OAD or LOAD. Hourly changes in **(A)** energy expenditure, **(C)** respiratory quotient, and **(E)** locomotor activity are shown over 24 h. Dark and light cycles are indicated by gray and white backgrounds, respectively. **(B)** Average energy expenditure, **(D)** respiratory quotient, and **(F)** locomotor activity in the dark and light cycles are shown. Data are expressed as means ± SEMs (n = 6 for each group).

We tested whether a two-week feeding of LOAD affects the biosynthesis of intestinal FAEs and food intake in mice. Jejunal FAE levels were measured in mice fed the OAD or LOAD for two weeks. As shown in **Figure 1A**, jejunal OEA levels were reduced by 50% in the LOAD group compared with those in the OAD group (P = 0.0104). In addition, jejunal OA levels were decreased by 73% in the LOAD group compared with the OAD group (P = 0.0317, **Figure 1G**). In contrast, AEA, PEA, and LEA levels were not affected by LOAD feeding (**Figures 1B–D**). There was no significant difference in jejunal SEA and SA levels between the groups (**Figures 1E, F**). Thus, reducing dietary OA levels specifically decreased jejunal OA and OEA levels. Next, we investigated whether reduced jejunal OEA levels, without changes to PEA and LEA levels, affect food intake and body weight gain. As shown in **Figure 2A**, food intake was increased in LOAD-fed mice compared with that of OAD-fed mice (P = 0.0177). Additionally, body weight gain was significantly increased in the LOAD group compared with that in the OAD group (P = 0.0266; **Figure 2C**), although the increase in final body weight was not significant (P = 0.0518, **Figure 2B**). The weights of epididymal (P = 0.0437) and peritoneal (P = 0.0131) white adipose tissues, but not those of the liver, cecum, subcutaneous adipose tissue, and brown adipose tissue, were increased in the LOAD group relative to those in the OAD group (**Figure 2D**). However, there was no difference in the blood glucose (**Figure 2E**), plasma triacylglycerol (**Figure 2F**), and cholesterol (**Figure 2G**) levels between the two groups. Energy expenditure, respiratory quotient, and locomotor activity did not differ in OAD- and LOAD-fed mice following two weeks of feeding (**Figure 3**). These data suggest that short-term feeding on the LOAD reduced intestinal OEA levels, resulting in increased food intake and body weight gain. Hydrogenated oils have lower digestibility than standard oils; thus, their consumption might lead to the reduced absorption of fat and energy (22, 23). Therefore, we examined the fecal excretion and digestibility of dietary fat in mice fed OAD and LOAD for two weeks. Fecal dry weight was increased 1.6-fold in the LOAD group compared with that in the OAD group (P < 0.001; **Figure 2H**). Fat excretion was also significantly higher in the LOAD group compared with that in the OAD group (P = 0.0161; **Figure 2I**), and the calculated digestibility coefficient was lower in the LOAD group (P = 0.0280; **Figure 2J**). Although fat excretion was increased in LOAD group, the energy absorbed at two weeks was significantly higher in the LOAD group compared with that in the OAD group (P = 0.0280; **Figure 2K**).

### 3.2 Effects of cyclic LOAD feeding

**Figure 4:**
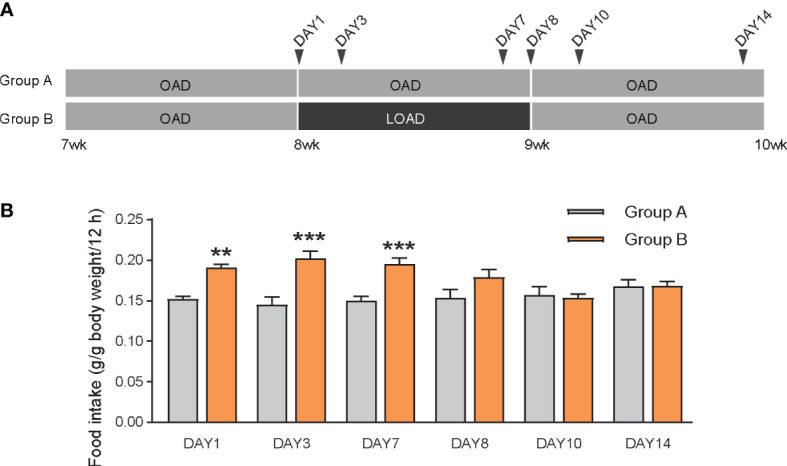
Food intake of mice during cyclic feeding on the OAD and LOAD. **(A)** Experimental design of the cyclic feeding experiment. **(B)** Food intake was measured on days 1, 3, 7, 8, 10 and 14 during two weeks feeding. Data are expressed as means ± SEMs (n = 7). **P < 0.01; ***P < 0.001.

We performed cyclic feeding of experimental diets to check whether the effects on food intake of the diets was induced by intestinal sensing of OA by mice. As shown in **Figure 4A**, seven-week-old mice in both groups were fed OAD for 1 week, after which group A was fed the OAD for an additional two weeks and group B switched to LOAD feeding for one week before switching back to OAD feeding for one additional week. Food intake during the dark periods was measured on day 1 (switching day from the OAD to the LOAD in group B), 3, 7, 8 (switching day from the LOAD to the OAD in group B), 10, and 14. Food intake was increased in group B on days 1 (P < 0.001), 3 (P = 0.0018), and 7 (P < 0.001) (**Figure 4B**); however, food intake did not differ between the groups on days 8, 10, and 14. These results indicate that the effects of dietary OA on food intake occurred immediately, likely due to the detection of the OA levels by the mice.

### 3.3 Effects of OA and OEA treatments on LOAD feeding

**Figure 5:**
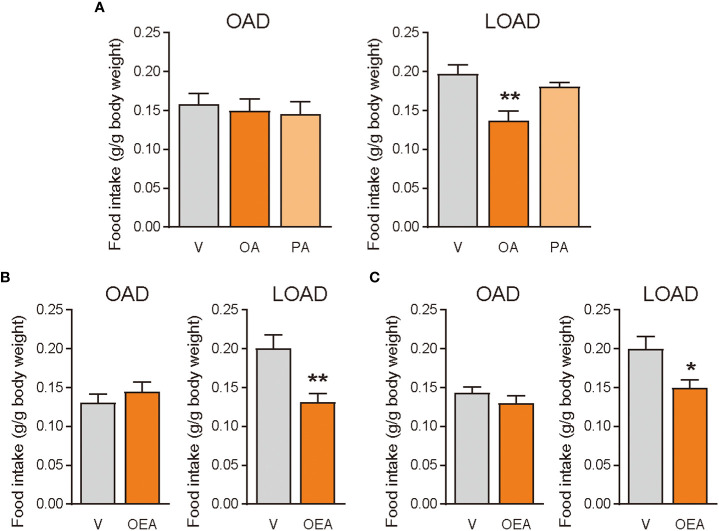
Effects of OA and OEA treatment on food intake in mice fed the OAD and LOAD. Mice were fed the OAD for 1 week and then treated with fatty acids and OEA 30 min before the dark cycle. They were fed either the OAD or LOAD during a 12 h period, and their food intake was measured. **(A)** In the LOAD group, oral gavage of OA (100 mg of OA/mouse) but not PA (100 mg of PA/mouse) reduced food intake compared with that observed following the vehicle treatment (V; PBS containing 0.3% xanthan gum). **(B)** Oral gavage of OEA (5 mg/mouse) reduced food intake in the LOAD group compared to that observed following the vehicle treatment (V; PBS containing 10% DMSO and 0.3% xanthan gum). **(C)** Intraperitoneal treatment of OEA (0.5 mg/mouse) reduced food intake in the LOAD group compare with that observed following the vehicle treatment (V; PBS containing 70% DMSO). Values are expressed as means ± SEMs (n = 5 for V; n = 6 for OA or OEA). *P < 0.05; **P < 0.01.

We tested whether treatments of OA (oral gavage) and OEA (oral gavage and intraperitoneal treatment) could diminish the effects of LOAD on food intake in mice on day 1 (**Figure 4A**). Oral gavage of OA (100 mg/mouse) but not of PA (100 mg/mouse) significantly reduced food intake in LOAD-fed mice (P = 0.0035; **Figure 5A**). However, OA did not affect the food intake of OAD-fed mice (**Figure 5A**). Oral gavage (5 mg of OEA/mouse; P = 0.0070; **Figure 5B**) and intraperitoneal treatment (0.5 mg of OEA/mouse; P = 0.0213; **Figure 5C**) with OEA reduced food intake in LOAD-fed mice, but these treatments did not affect the food intake of OAD-fed mice.

### 3.4 Effect of refeeding after food deprivation

**Figure 6:**
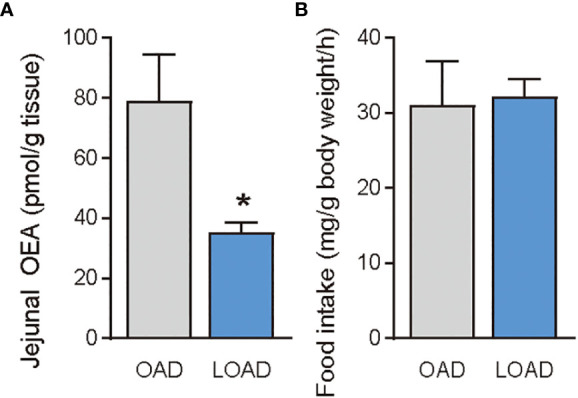
Jejunal OEA levels and food intake of mice fed the OAD and LOAD after 24 h of food deprivation. Mice were fed the OAD for one week and then deprived of food for 24 h. They were then fed either OAD or LOAD for 1 h. **(A)** Jejuna OEA levels and **(B)** food intake measured in mice. Values are expressed as means ± SEMs (n = 3). *P < 0.05.

Previous studies have shown that treatment of OEA strikingly reduced food intake in free-feeding rodents rather than re-feeding rodents after food deprivation (3). Therefore, we investigated whether OAD and LOAD feeding affects the intestinal OEA levels and food intake of mice refed after 24 h of food deprivation. As expected, intestinal OEA levels were significantly lower in the LOAD group compared with those in the OAD group (P = 0.0497), but there were no differences in the AEA, PEA, LEA, and SEA levels between the two groups (**Figure 6A** for OEA; other FAE data not shown) nor differences in their food intake (**Figure 6B**).

### 3.5 Effects of LOAD feeding for fifteen weeks

**Figure 7:**
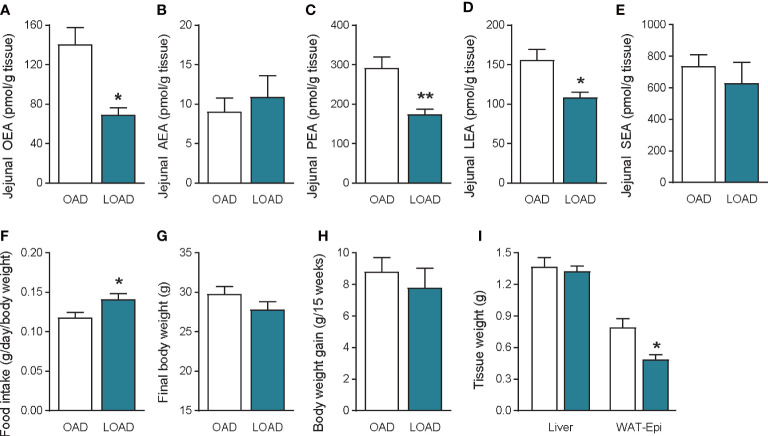
Jejunal FAE levels, food intake, body weight gain, and tissue weight in mice fed the OAD and LOAD for fifteen weeks. Jejunal FAE levels were measured using LC–MS/MS. **(A)** OEA, **(B)** AEA, **(C)** PEA, and **(D)** LEA, and **(E)** SEA levels. **(F)** Food intake was measured on the last day of the feeding period. **(G)** Final body weight on the last day of the feeding period. **(H)** Body weight gain during the feeding period. **(I)** Weight of the liver and epidydimal adipose tissue (WAT-Epi). Values are expressed as means ± SEMs (n = 4 or 5). *P < 0.05, **P < 0.01.

We investigated whether a fifteen-week feeding of LOAD affects jejunal OEA levels and food intake in mice. Similar to the two-week feeding period (**Figure 1A**), jejunal OEA levels were reduced by 50% in the LOAD group compared with those in the OAD group (P = 0.0103; **Figure 7A**). Although jejunal AEA (**Figure 7B**) and SEA (**Figure 7E**) levels did not differ in the two groups, jejunal PEA (P = 0.0093; **Figure 7C**) and LEA (P = 0.0246; **Figure 7D**) levels were reduced in the LOAD group compared with those in the OAD group. In association with the reduced levels of OEA, food intake was increased in the LOAD group (P = 0.0463; **Figure 7F**). However, the final body weight (**Figure 7G**) and body weight gain (**Figure 7H**) of mice did not differ between the two groups. The weight of the epididymal adipose tissue, but not that of the liver (**Figure 7I**), brain, heart, kidneys, spleen, testes, and lungs (data not shown), was reduced in the LOAD group. These results show that long-term LOAD feeding has effects on the food intake and jejunal OEA levels, which is similar to those observed following short-term LOAD feeding. On the other hand, long-term LOAD feeding exhibited different effects on the levels of jejunal PEA and LEA, body weight gain and fat accumulation compared to that of OAD.

## 4 Discussion

In this study, we investigated the importance of dietary OA in the regulation of food intake *via* intestinal OEA biosynthesis using a feeding experiment in which mice were fed two specific diets, the OAD and LOAD, which are based on AIN-93G (7% fat) (**Tables 1** and **2**). The OAD contained 38.4 mg of OA/g diet, which has been reported as a sufficient amount of OA for rodents (16, 17), whereas the LOAD contained only 3.1 mg of OA/g diet as well as a high amount of SA (42.4 mg/g diet) (**Table 2**). As expected, LOAD feeding reduced the jejunal OEA levels of mice compared with those in mice fed the OAD (**Figures 1**, **7**), resulting in increased food intake (**Figures 2**, **7**). The effects of LOAD feeding occurred immediately (**Figure 4**) and remained until week 15 of feeding; however, mice fed the LOAD for fifteen weeks exhibited differences in body weight gain, tissue weight, and other FAE levels compared with those of mice fed the LOAD for two weeks (**Figure 7**). We also found that the oral administration of OA and OEA attenuated the LOAD-mediated increase in food intake in mice. Taken together, these results highlighted significant impact of dietary OA in the regulation of food intake and satiety *via* intestinal sensing of dietary fat and OEA signaling.

Similar to OEA, LEA and PEA have been reported to control food intake. Administering PEA and LEA *via* intraperitoneal injection or oral gavage to rodents has been shown to reduce their food intake (11, 12, 24). Previous studies have also shown that refeeding and intestinal infusion of dietary fat increase intestinal OEA and LEA levels but not PEA levels (5, 6). Moreover, intestinal infusion of OA or linoleic acid are known to increase OEA or LEA levels, respectively, in rodents (6, 25). Thus, LEA synthesized in the intestine apparently induces satiety in a similar manner to OEA. The two diets used in this study contained similar levels of linoleic acid but different amounts of OA (**Table 2**). We found no difference in LEA levels between the OAD and LOAD groups after two weeks of feeding, but LOAD feeding led to reduced jejunal OEA levels compared with those detected following OAD feeding (**Figure 1**) as well as increased food intake and body weight gain (**Figure 2**), and these effects were attenuated by OA and OEA treatment (**Figure 5**). Therefore, intestinal OEA synthesized from dietary OA apparently controls food intake without the influence of intestinal LEA synthesized from dietary linoleic acid.

OA is not an essential fatty acid because it can be synthesized from SA. Stearoyl-CoA desaturase 1 (SCD1) is the rate limiting enzyme that catalyzes the biosynthesis of monounsaturated fatty acids from saturated fatty acids (26). SCD1 is expressed at high levels in the liver and adipose tissue but is also expressed in other tissues, including the intestine (27, 28). Although SCD1 is expressed in the intestine at relatively low levels, it plays important roles in the synthesis of OA in the enterocytes and the maintenance of fatty acid homeostasis (15, 27). Conditional SCD1–knockout mice with knockout in the intestinal epithelium exhibited inflammation and tumor burden in the gut, but OA feeding reversed these effects (15). Therefore, the intestine is apparently capable of synthesizing OA from SA, and the synthesized OA can maintain physiological activities. Although we cannot exclude the possibility of OA synthesis from SA in the intestines, LOAD feeding resulted in lower jejunal OEA concentrations and increased food intake regardless of the feeding duration up to 15 weeks. Thus, we suggest that, although dietary OA is not an essential fatty acid, it can be defined as conditionally essential to regulate food intake and satiety (16, 17).

Fifteen-week feeding of LOAD increased food intake and reduced jejunal OEA in mice (**Figure 7**), which is similar to those observed following two-week feeding of LOAD (**Figures 1** and **2**). On the other hand, fifteen-week feeding of LOAD showed different effects on the levels of jejunal PEA and LEA, body weight gain and fat accumulation compared to that of two-week feeding (**Figures 1**, **2** and **7**). This difference may be due to metabolic and molecular adaptations in mice raised under low OA conditions for fifteen weeks, although intestinal OA sensing was still functional. In experiments with rodents, long-term feeding is often used to obtain adaptations to diets lacking specific fatty acids (29–32). Sufficient levels of OA might be synthesized from SA in the organs such liver in the mice fed LOAD for fifteen weeks because plasma palmitoleic acid was not changed between the OAD and LOAD groups (data not shown), suggesting metabolic and molecular adaptations of mice to the diet. Detailed investigations are needed to reveal metabolic change and molecular events under prolonged low OA condition.

Clinical studies have shown that dietary OA levels and intraduodenal OA infusion affect subsequent food intake in humans. For example, Menella et al. (33) suggested that feeding on experimental meals containing higher OA levels (80% of total fatty acid vs. 10% linoleic acid) led to a substantial reduction in energy intake and appetite compared with those detected following feeding on low OA meals (30% of total fatty acids vs. 55% linoleic acid). The authors also found that the plasma levels of OEA, but not those of LEA and PEA, were higher in the subjects fed the high-OA experimental meals. Stewart et al. (34) reported that intraduodenal infusion of OA substantially reduced the energy intake of lean subjects and that sensitivity to OA was lower in obese subjects. These results suggest that OEA-mediated signaling induced by dietary OA also occurs in the human intestine. Therefore, in addition to conducting clinical trials to investigate OEA as a satiety control agent (35, 36), studies should be performed to determine the importance of dietary OA in the OEA-mediated induction of satiety in humans.

In conclusion, this study provides novel demonstration that dietary OA has an impact in the reduction of food intake and increase in satiety mediated by OEA signaling. Although further studies are required, dietary OA may be conditionally essential for the feeding regulation (16, 17). Furthermore, these findings may offer new concepts of nutritional balance in the diet and new foods for regulating energy intake using oleic acid-containing oils.

## Data availability statement

The raw data supporting the conclusions of this article will be made available by the authors, without undue reservation.

## Ethics statement

The animal study was reviewed and approved by The Committee on the Ethics of Animal Experiments of Saitama Medical University and the Tokyo University of Agriculture and Technology.

## Author contributions

MI, conceptualization, data curation, funding acquisition, investigation, and writing – original draft. KI, TH, and TT, investigation. IK and KM, resources. KY, conceptualization and data curation. All, writing – review and editing. All authors contributed to the article and approved the submitted version.
